# Efficacy and safety of aclidinium bromide/formoterol fumarate fixed-dose combinations compared with individual components and placebo in patients with COPD (ACLIFORM-COPD): a multicentre, randomised study

**DOI:** 10.1186/1471-2466-14-178

**Published:** 2014-11-18

**Authors:** Dave Singh, Paul W Jones, Eric D Bateman, Stephanie Korn, Cristina Serra, Eduard Molins, Cynthia Caracta, Esther Garcia Gil, Anne Leselbaum

**Affiliations:** University of Manchester, Medicines Evaluation Unit, University Hospital of South Manchester NHS Foundation Trust, Langley Building, Southmoor Road, Manchester, M23 9QZ UK; Infection and Immunity Institute, St George’s, University of London, London, UK; Division of Pulmonology, Department of Medicine, University of Cape Town, Cape Town, South Africa; Pulmonary Department, Mainz University Hospital, Mainz, Germany; Almirall R&D Centre, Almirall, Barcelona, Spain; AstraZeneca PLC, Barcelona, Spain; Formerly of Forest Research Institute, Forest Laboratories LLC, a subsidiary of Actavis, Jersey City, NJ USA; Formerly of Almirall S.A., Barcelona, Spain

**Keywords:** Aclidinium bromide/formoterol fumarate, Chronic obstructive pulmonary disease, Fixed-dose combination

## Abstract

**Background:**

Aclidinium/formoterol is a twice-daily (BID) fixed-dose combination (FDC) in development for chronic obstructive pulmonary disease (COPD). The efficacy and safety of aclidinium/formoterol versus monotherapy and placebo in patients with COPD was assessed.

**Methods:**

In this 24-week double-blind, parallel-group, active- and placebo-controlled, multicentre Phase III study, patients (≥40 years, post-bronchodilator forced expiratory volume in 1 second [FEV_1_]/forced vital capacity <70% and FEV_1_ ≥30% but <80% predicted normal) were randomised 2:2:2:2:1 to aclidinium/formoterol 400/12 μg (n = 385) or 400/6 μg (n = 381), aclidinium 400 μg (n = 385), formoterol 12 μg (n = 384) or placebo (n = 194) BID via Genuair^®^/Pressair^®a^.

**Results:**

At Week 24, aclidinium/formoterol 400/12 μg and 400/6 μg lead to significant improvements from baseline in 1-hour post-dose FEV_1_ versus aclidinium (125 mL [95% CI: 90, 160; p < 0 · 001] and 69 mL [95% CI: 34, 105; p < 0.001], respectively) and trough FEV_1_ versus formoterol (85 mL [95% CI: 51, 119; p < 0.001] and 53 mL [95% CI: 19, 87; p < 0.01], respectively; co-primary endpoints). Additionally, aclidinium/formoterol 400/12 μg and 400/6 μg provided significant improvements in Transition Dyspnoea Index (TDI) focal score versus placebo (1.29 units [95% CI: 0.73, 1.86; p < 0.001] and 1.16 units [95% CI: 0.59, 1.73; p < 0.001], respectively; secondary endpoint). All treatments were well tolerated, with safety profiles of the FDCs similar to those of placebo and monotherapy.

**Conclusions:**

Both aclidinium/formoterol BID doses significantly improved bronchodilation versus monotherapy, and dyspnoea versus placebo, with no increase in safety risk. Aclidinium/formoterol may be an effective treatment for patients with COPD.

**Trial registration:**

ClinicalTrials.gov: NCT01462942.

**Electronic supplementary material:**

The online version of this article (doi:10.1186/1471-2466-14-178) contains supplementary material, which is available to authorized users.

## Background

Inhaled long-acting bronchodilators (long-acting β_2_-agonists [LABAs] and long-acting muscarinic antagonists [LAMAs]) are recommended as the first choice of treatment for patients with symptomatic chronic obstructive lung disease (COPD) [1]. LAMAs and LABAs relax airway smooth muscle by different mechanisms of action, and when combined may cause greater bronchodilation than a single agent [2, 3]. A combined LABA/LAMA is a recommended treatment option in patients with moderate-to-severe COPD [1]. Although a LAMA and LABA can be administered together using separate inhalers (i.e. as a ‘free combination’), this usage is not common, probably due to the inconvenience of handling separate devices [4]; delivery of medication with a single device is always more convenient for the patient. This has prompted the development of fixed-dose combinations (FDCs) of LAMAs and LABAs, such as glycopyrronium/indacaterol and umeclidinium/vilanterol, with the additional potential benefit of improved adherence to therapy [5].

Unlike many other LAMA/LABA FDCs, the combination of the LAMA aclidinium bromide and the LABA formoterol fumarate is administered twice daily (BID) [5–7]. BID dosing may provide a 24-hour profile of lung function improvements that has the potential to improve the night-time, early morning and day-time symptoms of COPD that are common in patients with this condition [7, 8].

This Phase III, randomised study aimed to compare bronchodilator efficacy, effects on symptoms and health status, and safety of two doses of aclidinium/formoterol FDC (400/12 μg and 400/6 μg) BID versus the monotherapy components (aclidinium 400 μg and formoterol 12 μg) and placebo over six months in patients with stable, moderate-to-severe COPD.

## Methods

### Study design

The ACLIFORM-COPD study (ClinicalTrials.gov NCT01462942) was a double-blind, randomised, parallel-group, active- and placebo-controlled, multicentre study conducted at 193 centres in 22 countries (see Additional file 1). The first patient enrolled on 26 October 2011; the last patient completed 4 January 2013.

Following screening and a 2–3-week run-in period, patients with stable, moderate-to-severe COPD were randomised 2:2:2:2:1 to 24 weeks of double-blind treatment with twice-daily aclidinium/formoterol FDC 400/12 μg or 400/6 μg, aclidinium 400 μg, formoterol 12 μg or placebo, all via a breath-actuated, multiple-dose dry powder inhaler (Genuair^®^/Pressair^®a^; Almirall S.A., Barcelona, Spain).

This study was conducted in accordance with the Declaration of Helsinki, International Conference on Harmonisation/Good Clinical Practice Guidelines, and local regulations. The protocol was approved by the regulatory authority for each country and an independent ethics committee at each centre (Additional file 1: Table S1). Patients gave written informed consent.

### Randomisation and masking

Randomisation was performed using a centralised interactive voice response system on Day 1 (Visit 1), with stratification by smoking status (smoker or ex-smoker). Patients were instructed to administer one puff of study treatment at the same time in the morning (8:00–10:00 am) and evening (8:00–10:00 pm). Treatment identity was concealed with identical packaging/appearance and no odour or colour. Patients received training to use Genuair^®^/Pressair^®^ at screening and Visit 1.

### Study population and concomitant medication

Male and female patients ≥40 years of age who were current or former cigarette smokers with a smoking history ≥10 pack-years and diagnosed with moderate-to-severe COPD according to GOLD 2010 criteria [9] (post-bronchodilator forced expiratory volume in 1 second (FEV_1_)/forced vital capacity (FVC) <70% and FEV_1_ ≥30% but <80% of predicted normal value) were eligible for inclusion (see Additional file 1 for exclusion criteria).

Inhaled salbutamol (100 μg/puff) was permitted as relief medication as needed, but discontinued 6 hours before planned study visits. Patients could continue inhaled corticosteroids (ICS) provided treatment was stable ≥4 weeks pre-screening (see Additional file 1 for additional concomitant medications).

### Assessments

Study assessments at each visit are outlined in Table 1. Spirometry (FEV_1_) was performed pre-dose and up to 3 hours post-dose, with additional spirometry measurements performed in a subset of patients (12-hour spirometry sub-study; ~20% of the intent-to-treat [ITT] population).

**Table 1:** **Timeline of study assessments**

	Run-in	Double-blind treatment						Follow-up
	Week -3 to -2	Day 1	Week 1	Week 4	Week 12	Week 18	Week 24	Week 26
Assessment	(Screening)	(Visit 1) ^a^	(Visit 2)	(Visit 3)	(Visit 4)	(Visit 5)	(Visit 6)	(Visit 7)
Pre-dose spirometry^b^		X	X	X	X	X	X	
Post-dose spirometry^c^		X^d^	X	X	X		X	
BDI/TDI^e^ and SGRQ		X		X	X		X	
EXACT^f^	X	X	X	X	X	X	X	
NT and EM symptoms^g^	X	X	X	X	X	X	X	
COPD exacerbations		X	X	X	X	X	X	X
12-hour spirometry sub-study^h^		X			X		X	
AEs	X	X	X	X	X	X	X	X
Laboratory tests (fasted)	X				X^i^		X^i^	
ECG and blood pressure	X	X^j^	X^j^	X^j^	X^j^		X^j^	
24-hour Holter sub-study	X				X^k^		X^k^	

^a^Randomisation visit; ^b^Two sets of manoeuvres were performed during the hour pre-morning-dose; ^c^One set of manoeuvres was performed at 0.5, 1, 2, and 3 hours post-morning-dose; ^d^An additional set of manoeuvres was performed at 5 minutes post-morning-dose; ^e^BDI was used at Day 1, TDI at all other visits; ^f^EXACT was completed every evening; ^g^NT and EM symptoms were recorded every morning; ^h^Additional spirometry measurements at 4, 6, 8, 10, and 12 hours post-morning-dose were performed in a sub-set of patients; ^i^Blood samples were taken pre-morning-dose; ^j^ECG and blood pressure were measured pre- and 2-hours post-morning-dose; ^k^24-hour Holter recordings were started from 5 minutes pre-dose on the morning prior to these visits.

AE, adverse event; BDI, Baseline Dyspnoea Index; COPD, chronic obstructive pulmonary disease; ECG, electrocardiogram; EM, early morning; EXACT, EXAcerbations of Chronic obstructive pulmonary disease Tool; NT, night-time; SGRQ, St George’s Respiratory Questionnaire; TDI, Transition Dyspnoea Index.

Dyspnoea was assessed at baseline using the Baseline Dyspnoea Index (BDI), with changes measured using the Transition Dyspnoea Index (TDI). Health status was assessed using St George’s Respiratory Questionnaire (SGRQ). COPD symptoms (day-time, night-time and early morning) and relief medication use were recorded in an electronic diary. Day-time symptoms were assessed using the EXAcerbations of Chronic obstructive pulmonary disease Tool (EXACT), a 14-item diary completed every night [10, 11]. Additionally, changes in specific respiratory symptoms (breathlessness, cough and sputum, and chest symptom domains) were assessed using the EXACT-Respiratory Symptoms score (E-RS; consisting of 11 items of the 14-item EXACT) [12, 13]. Night-time and early morning symptoms were assessed using a 14-item questionnaire completed every morning. COPD exacerbations were additionally assessed by Healthcare Resource Utilisation (HCRU) (see Additional file 1 for further details of symptoms assessments).

Safety assessments included recording of adverse events (AEs), laboratory tests, blood pressure measurements, 12-lead electrocardiograms (ECGs) and 24-hour 12-lead Holter recordings (sub-study, ~20% of the safety population). Major adverse cardiovascular events (MACE; a composite of total cardiovascular death, non-fatal myocardial infarction and non-fatal stroke) were evaluated and classified by an independent, blinded adjudication committee.

### Endpoints

The co-primary endpoints were change from baseline at Week 24 in 1-hour morning post-dose FEV_1_ versus aclidinium 400 μg and morning pre-dose (trough) FEV_1_ versus formoterol 12 μg; these endpoints were specified based on European Medicines Agency guidelines and US Federal Drug Administration regulations, which state that each drug in a fixed-dose combination must make a documented contribution within the combination to the claimed effects [14, 15]. Secondary endpoints were TDI focal score and change from baseline in SGRQ total score at Week 24 (both versus placebo). Additional efficacy endpoints were change from baseline in FEV_1_ during 3 hours post-dose, peak FEV_1_, TDI and SGRQ responders (% patients achieving the minimum clinically important difference [MCID] in TDI [≥1 unit increase] and SGRQ [≥4 units]); changes from baseline in COPD symptoms (total EXACT-Respiratory Symptoms [E-RS] score, and night-time and early morning symptoms scores); rate of COPD exacerbations (HCRU and EXACT); and change from baseline in relief medication use.

### Statistical analysis

Data were analysed using SAS^®^ Version 9.3. Efficacy analyses, with the exception of exacerbation rate, were performed on the ITT population (patients who took ≥1 dose of study medication and had a baseline and ≥1 post-baseline FEV_1_ assessment). Exacerbations and safety outcomes were assessed in the safety population (patients who received ≥1 dose of study medication).

A sample size of 1575 (350 per active treatment; 175 placebo) was estimated to provide ≥90% power to detect a significant difference of 100 mL between aclidinium/formoterol FDC and aclidinium in change from baseline in 1-hour post-dose FEV_1_ (with standard deviation [SD] 280 mL); 65 mL between aclidinium/formoterol FDC and formoterol 12 μg in change from baseline in trough FEV_1_ (with SD 260 mL); and ≥1 unit difference in TDI focal score (with SD 3.4 units) and ≥4 unit difference in SGRQ total score (with SD 12.8 units) between aclidinium/formoterol FDC and placebo at Week 24 using two-sided tests and adjusting for multiple tests at the overall significance level of 0.05.

Pulmonary function variables, COPD symptoms (including TDI focal score), SGRQ total score and relief medication use were analysed by means of a mixed model for repeated measures (MMRM), adjusted by age and baseline value as covariates, and treatment group, sex, smoking status, visit and treatment group-by-visit interaction as fixed-effect factors. The MMRM for FEV_1_ variables was additionally adjusted by screening pre- and post-bronchodilator (salbutamol) FEV_1_ as a covariate. Efficacy variable treatment effects and treatment differences were estimated by least squares (LS) means. Safety outcomes were analysed descriptively for the safety population. Additionally, regression analyses were performed for exacerbation rate (see Additional file 1).

## Results

**Figure 1:**
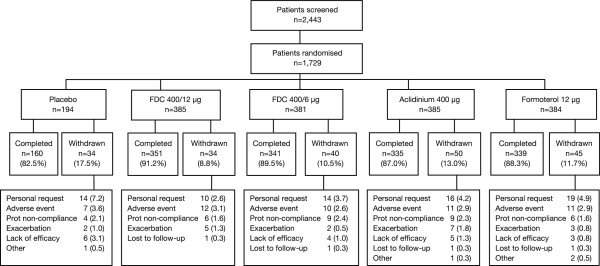
**Patient disposition.** AE, adverse event; FDC, fixed-dose combination of aclidinium/formoterol; prot, protocol.

Of 2443 patients screened, 1729 were included in the safety analysis set; three patients were excluded from the ITT population due to missing FEV_1_ data (Figure 1). In total, 203 (11.7%) patients discontinued treatment; more patients in the placebo group withdrew prematurely (17.5%) compared with active treatments (8.8–13.0%). The primary reasons for withdrawal were patient request (4.2%), AEs other than COPD exacerbation (2.9%) and protocol non-compliance (2.0%).

Demographics and baseline characteristics were similar across treatment groups (Table 2). Post-bronchodilator FEV_1_ was approximately 55% in all treatment groups. Prior to the study, 19.8% of patients were using ICS as monotherapy (or in free combination) and 31.8% of patients were using ICS in fixed-dose combination with a LABA (Table 2).

**Table 2:** **Patient demographics and baseline characteristics (safety population)**

	Placebo	FDC	FDC	Aclidinium	Formoterol	Total
		400/12 μg	400/6 μg	400 μg	12 μg	
Patients, n	194	385	381	385	384	1729
Age, years	64.2 ± 8.0	62.7 ± 8.1	62.9 ± 7.7	63.1 ± 8.2	63.4 ± 7.8	63.2 ± 8.0
Males, n (%)	138 (71.1)	261 (67.8)	259 (68.0)	256 (66.5)	255 (66.4)	1169 (67.6)
Caucasians, n (%)	183 (94.3)	367 (95.3)	366 (96.1)	363 (94.3)	362 (94.3)	1641 (94.9)
Current smoker, n (%)	94 (48.5)	181 (47.0)	182 (47.8)	182 (47.3)	179 (46.6)	818 (47.3)
Severity of airflow obstruction, n (%), based on GOLD 2010 criteria^a^						
Moderate	116 (60.1)	229 (59.5)	230 (60.4)	226 (58.9)	237 (61.9)	1038 (60.1)
Severe	77 (39.9)	156 (40.5)	151 (39.6)	157 (40.9)	144 (37.6)	685 (39.7)
Baseline FEV_1_, L	1.42 ± 0.54	1.42 ± 0.49	1.41 ± 0.48	1.40 ± 0.51	1.40 ± 0.48	1.41 ± 0.50
Post-bronchodilator FEV_1_, % predicted	55.0 ± 13.4	54.6 ± 13.1	54.1 ± 13.0	53.6 ± 13.0	54.5 ± 13.2	54.3 ± 13.1
Patients meeting bronchial reversibility criteria, n (%)^b^	66 (34.0)	134 (34.8)	127 (33.3)	125 (32.6)	114 (29.8)	566 (32.8)
Prior COPD medication, n (%)						
Any COPD-related medication	167 (86.1)	330 (85.7)	331 (86.9)	337 (87.5)	329 (85.7)	1494 (86.4)
LABA + ICS^c^	63 (32.5)	118 (30.6)	128 (33.6)	121 (31.4)	119 (31.0)	549 (31.8)
LAMA	60 (30.9)	118 (30.6)	122 (32.0)	107 (27.8)	110 (28.6)	517 (29.9)
LABA	38 (19.6)	79 (20.5)	69 (18.1)	78 (20.3)	80 (20.8)	344 (19.9)
ICS	39 (20.1)	85 (22.1)	72 (18.9)	79 (20.5)	68 (17.7)	343 (19.8)
BDI focal score	6.6 ± 2.0	6.6 ± 2.1	6.6 ± 2.0	6.5 ± 2.0	6.5 ± 2.1	6.5 ± 2.1
SGRQ total score	45.8 ± 17.6	46.1 ± 17.9	46.7 ± 17.6	46.8 ± 16.8	45.2 ± 18.2	46.2 ± 17.6

^a^One patient randomised to formoterol 12 μg had mild COPD at baseline; one patient each randomised to formoterol 12 μg and aclidinium 400 μg had very severe COPD at baseline; ^b^Bronchial reversibility ≥12% and change in FEV_1_ from pre-test ≥200 mL; ^c^LABA and ICS in fixed-dose combination.

Data are presented as mean ± SD, unless otherwise stated.

BDI, Baseline Dyspnoea Index; COPD, chronic obstructive pulmonary disease; FDC, aclidinium/formoterol fixed-dose combination; FEV_1,_ forced expiratory volume in 1 second; GOLD, Global Initiative for Chronic Obstructive Lung Disease; ICS, inhaled corticosteroid; LABA, long-acting β_2_-adrenergic agonist; LAMA, long-acting muscarinic antagonist; SD, standard deviation; SGRQ, St George’s Respiratory Questionnaire.

### Pulmonary function

**Figure 2:**
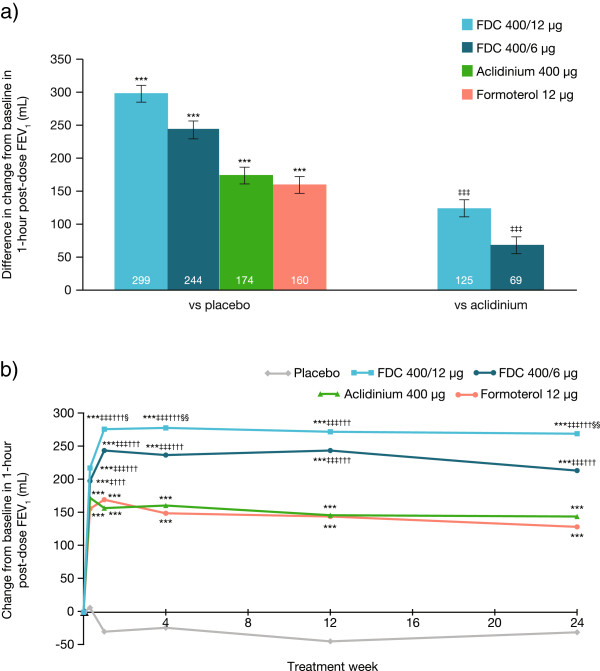
**Mean treatment differences for change from baseline in 1-hour post-dose FEV**_**1**_ **. (a)** At Week 24; **(b)** Over 24 weeks; Data are presented as least squares means (SE) for the ITT population. ***p < 0.001 vs placebo; ^‡^p < 0.05; ^‡‡‡^p < 0.001 vs aclidinium; ^†††^p < 0.001 vs formoterol; ^§^p < 0.05; ^§§^p < 0.01 vs FDC 400/6 μg. FDC, aclidinium/formoterol fixed-dose combination; FEV_1_, forced expiratory volume in 1 second; ITT, intent-to-treat; SE, standard error.

At Week 24, aclidinium/formoterol FDC 400/12 μg and 400/6 μg caused significantly greater changes from baseline in 1-hour morning post-dose FEV_1_ compared with placebo (LS means: 299 mL and 244 mL, respectively; both p < 0.001; Figure 2a). Aclidinium and formoterol monotherapies also caused improvements compared with placebo (LS means: 174 mL and 160 mL, respectively; both p < 0.001; Figure 2a). Figure 2b shows that these FEV_1_ changes were significantly greater with both FDC 400/12 μg and 400/6 μg versus either monotherapy at all timepoints (LS means at Week 24: 125 mL and 69 mL, respectively, versus aclidinium [both p < 0.001]; 139 mL and 84 mL versus formoterol [both p < 0.001]); the 400/12 μg dose was statistically superior to the 400/6 μg dose at all visits except Week 12 (all p < 0.05).

**Figure 3:**
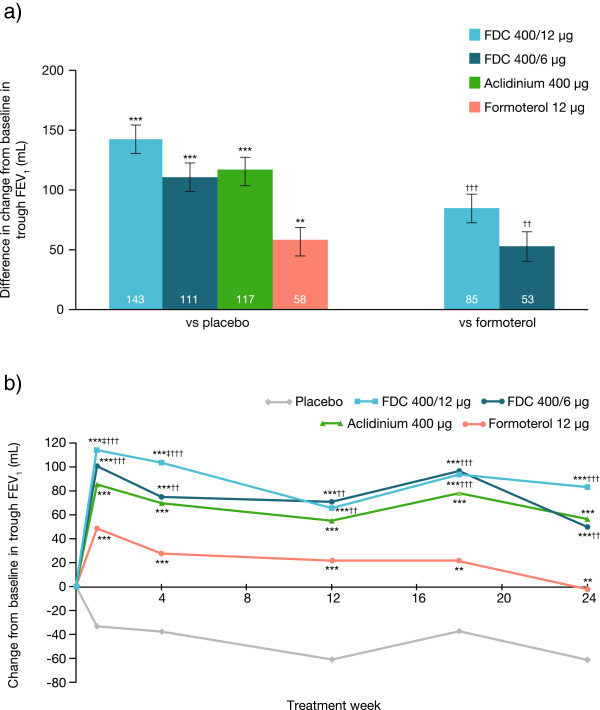
**Mean treatment differences for change from baseline in trough FEV**_**1**_ **. (a)** At Week 24; **(b)** Over 24 weeks; Data are presented as least squares means (SE) for the ITT population. **p < 0.01; *** p < 0.001 vs placebo; ^‡^p < 0.05 vs aclidinium; ^††^p < 0.01; ^†††^p < 0.001 vs formoterol. FDC, aclidinium/formoterol fixed-dose combination of aclidinium/formoterol; FEV_1_, forced expiratory volume in 1 second; ITT, intent-to-treat; SE, standard error.

Aclidinium/formoterol FDC 400/12 μg and 400/6 μg caused significantly greater changes from baseline in trough FEV_1_ at Week 24 compared with placebo (LS means: 143 mL and 111 mL, respectively; both p < 0.001; Figure 3a). Aclidinium and formoterol monotherapies also caused improvements compared with placebo (LS means: 117 mL [p < 0.001] and 58 mL [p < 0.01], respectively; Figure 3a). Figure 3b shows that these FEV_1_ changes were significantly greater with FDC 400/12 μg and 400/6 μg versus formoterol at all timepoints (LS means at Week 24: 85 mL [p < 0.001] and 53 mL [p < 0.01], respectively). Compared with aclidinium, improvements were numerically greater with FDC 400/12 μg at all visits and significantly greater at Weeks 1 and 4.

The onset of action of both FDC doses on Day 1 was fast, with significant improvements in bronchodilation versus placebo at 5 minutes post-dose (Additional file 1: Figure S1). The change in FEV_1_ over 3 hours post-dose, peak FEV_1_ and results of 12-hour spirometry are presented in Additional file 1. Generally, significant improvements in these parameters were observed with FDC at all visits compared with placebo and one or both monotherapies.

### Symptoms

**Figure 4:**
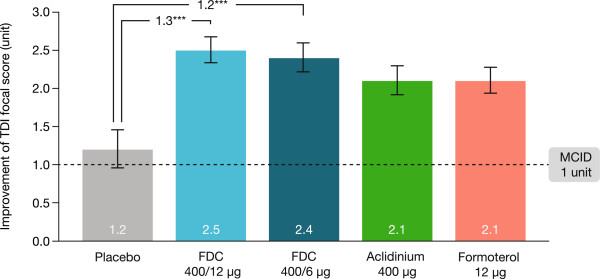
**Improvement in TDI focal score at 24 weeks (ITT population).** Data are presented as least squares means (SE). ^***^p < 0.001 vs placebo. FDC, aclidinium/formoterol fixed-dose combination; ITT, intent-to-treat; MCID, minimum clinically important difference; SE, standard error; TDI, transition dyspnoea index.

At Week 24, both FDC doses caused clinically significant improvements (≥1 unit) in TDI focal score versus placebo (400/12 μg: 1.29 units and 400/6 μg: 1.16 units; both p < 0.001; Figure 4). Aclidinium and formoterol monotherapies caused significant improvements (both p < 0.005) versus placebo at Week 24 that fell just below the 1-unit threshold. The differences between both FDC doses versus the monotherapies were not statistically significant (Additional file 1: Table S2). A significantly higher proportion of FDC-treated and monotherapy-treated patients had ≥1 unit improvement in TDI focal score at Week 24 versus placebo (Additional file 1: Table S2).

The improvements in overall E-RS symptoms with both FDC doses were significantly greater compared with the monotherapies and placebo (all comparisons p < 0.05); changes from baseline in night-time and early morning symptoms scores followed a similar pattern, although not all comparisons reached statistical significance (see Additional file 1).

COPD exacerbations data are presented in Additional file 1. The rate of HCRU exacerbations was low across treatment arms (0.26–0.41 per patient per year) and although the rate was numerically lower with both FDC doses compared with placebo, this difference was not statistically significant. When assessed with EXACT, higher exacerbation rates were observed (1.09–1.54 per patient per year) and the FDC 400/12 μg dose significantly reduced the rate of exacerbations compared with placebo (see Additional file 1: Table S3). During the 24 treatment weeks, the number of patients hospitalised due to exacerbations (based on HCRU data) was low and similar between treatment groups (placebo: n = 5 [2.6%]; FDC 400/12 μg: n = 4 [1.0%]; FDC 400/6 μg: n = 3 [0.8%]; aclidinium 400 μg: n = 7 [1.8%]; formoterol 12 μg: n = 1 [0.3%]).

### Health-related quality of life

**Figure 5:**
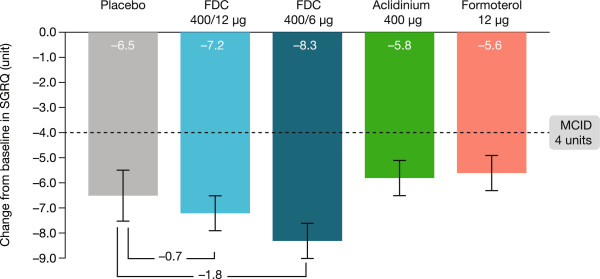
**Change from baseline in SGRQ total score at 24 weeks (ITT population).** Data are presented as least squares means (SE). FDC, aclidinium/formoterol fixed-dose combination; ITT, intent-to-treat; MCID, minimum clinically important difference; SE, standard error; SGRQ, St George’s Respiratory Questionnaire.

At Week 24, all active treatments were associated with improvements in mean SGRQ total score >4 units (Figure 5); however, there was a very high placebo response and there were no statistically significant differences between active and placebo treatments (Additional file 1: Table S2). A significantly higher proportion of patients receiving FDC 400/6 μg had ≥4 units decrease in SGRQ total score at Week 24 versus placebo (see Additional file 1).

### Relief medication use

Mean treatment differences in overall daily use of relief medication versus placebo were -0.66 with FDC 400/12 μg (p < 0.001) and -0.73 with 400/6 μg (p < 0.001). These changes from baseline with both FDC doses were significantly greater than aclidinium monotherapy (both p < 0.05), but not compared with formoterol monotherapy.

### Safety

The incidence of treatment-emergent AEs (TEAEs) was similar across study arms (Table 3, also see Additional file 1). The proportion of patients experiencing a serious AE (SAE) was low (4.8%) and comparable between treatment groups (Table 3). The most frequently reported SAE was COPD exacerbation (Table 3).

**Table 3:** **Number (%) of patients with TEAEs, TESAEs and discontinuations (safety population)**

		FDC	FDC	Aclidinium	Formoterol
	Placebo	400/12 μg	400/6 μg	400 μg	12 μg
n (%)	(n = 194)	(n = 385)	(n = 381)	(n = 385)	(n = 384)
Any TEAE	103 (53.1)	194 (50.4)	193 (50.7)	190 (49.4)	217 (56.5)
TEAEs in ≥2% of any treatment group (by preferred term)					
COPD exacerbation	27 (13.9)	36 (9.4)	38 (10.0)	46 (11.9)	60 (15.6)
Headache	16 (8.2)	29 (7.5)	27 (7.1)	35 (9.1)	43 (11.2)
Nasopharyngitis	14 (7.2)	30 (7.8)	30 (7.9)	22 (5.7)	26 (6.8)
Back pain	9 (4.6)	18 (4.7)	13 (3.4)	20 (5.2)	19 (4.9)
Diarrhoea	5 (2.6)	6 (1.6)	5 (1.3)	2 (0.5)	6 (1.6)
Nausea	5 (2.6)	3 (0.8)	4 (1.0)	4 (1.0)	1 (0.3)
Abdominal pain, upper	4 (2.1)	2 (0.5)	3 (0.8)	4 (1.0)	6 (1.6)
Arthralgia	3 (1.5)	6 (1.6)	3 (0.8)	3 (0.8)	12 (3.1)
URTI	3 (1.5)	8 (2.1)	4 (1.0)	7 (1.8)	10 (2.6)
Hypertension	2 (1.0)	3 (0.8)	4 (1.0)	2 (0.5)	9 (2.3)
Sinusitis	1 (0.5)	3 (0.8)	10 (2.6)	3 (0.8)	3 (0.8)
Rhinitis	1 (0.5)	2 (0.5)	6 (1.6)	2 (0.5)	10 (2.6)
Oropharyngeal pain	1 (0.5)	10 (2.6)	2 (0.5)	5 (1.3)	2 (0.5)
TEAEs leading to discontinuation	8 (4.1)	16 (4.2)	12 (3.1)	17 (4.4)	14 (3.6)
TESAEs	12 (6.2)	23 (6.0)	18 (4.7)	16 (4.2)	14 (3.6)
TESAEs occurring in >2 patients in any treatment group (by preferred term)					
COPD exacerbation	5 (2.6)	4 (1.0)	4 (1.0)	7 (1.8)	1 (0.3)
Pneumonia	1 (0.5)	3 (0.8)	4 (1.0)	0 (0.0)	0 (0.0)

COPD, chronic obstructive pulmonary disease; FDC, aclidinium/formoterol fixed-dose combination; TEAE, treatment-emergent adverse event; TESAE, treatment-emergent serious adverse event; URTI, upper respiratory tract infection.

The incidence of MACE was low and comparable across all study arms (see Additional file 1). TEAEs associated with anticholinergic or β_2_-adrenergic activity generally occurred in <3% of patients in any treatment group. The exception was headache (β_2_-adrenergic TEAE), reported in 7.1%–11.2% of patients. There were four fatal TEAEs (see Additional file 1); none were considered related to study medication. There were no clinically significant differences between treatment groups in clinical laboratory tests, vital signs and ECGs (including 24-hour Holter ECG monitoring; Additional file 1: Table S4).

There were four fatal TEAEs: one in the aclidinium/formoterol 400/12 μg group (COPD exacerbation), two in the aclidinium/formoterol 400/6 μg group (COPD exacerbation [n = 1] and cardiac failure [n = 1]) and one in the formoterol 12 μg group (cardiac failure). None of these were considered to be related to study medication and all four patients had multiple underlying comorbidities including prior cardiac failure, coronary artery disease and hypertension.

## Discussion

Both doses of aclidinium/formoterol FDC BID significantly improved pulmonary function after 24 weeks compared to the monotherapy components and placebo in patients with moderate-to-severe COPD. The greatest improvements were seen with the higher aclidinium/formoterol FDC dose. Clinical benefits with aclidinium/formoterol FDC were achieved without an increased risk of AEs.

The pre-defined co-primary endpoints in this study were chosen to test the individual contributions of the component therapies, i.e. rapid-onset bronchodilation with formoterol (change from baseline in FEV_1_ at 1-hour post-morning dose) [5, 16, 17], and 24-hour bronchodilation with twice-daily aclidinium (change from baseline in trough FEV_1_) [18, 19]. Both doses of aclidinium/formoterol FDC met both co-primary endpoints, suggesting that aclidinium/formoterol FDC provides bronchodilation that is faster in onset than aclidinium and of a greater magnitude over the dosing interval than formoterol. To date, all LAMA/LABA FDCs have demonstrated superiority to their monocomponents for improvements in FEV_1,_ and our study shows similar results [20–24]. Taken together, the results provide good evidence that LAMA/LABA FDCs deliver additional clinical benefits versus monotherapy. Currently, there is no established MCID in bronchodilation for combination therapies versus their monotherapy components [25]. In this study, the treatment differences versus monotherapy were generally greater with the 400/12 μg dose compared with the 400/6 μg dose for bronchodilation endpoints (change from baseline in 1-hour post-dose FEV_1_, trough and peak FEV_1_, FEV_1_ over 3 hours post-dose and normalised FEV_1_ AUC_0-12_).

Both aclidinium/formoterol FDC doses improved trough FEV_1_ by >100 mL compared with placebo; this threshold is a clinically meaningful change [26]. The contribution of aclidinium to the increase from baseline in trough FEV_1_ seen with aclidinium/formoterol 400/12 μg (85 mL versus formoterol) is within the range observed for the monocomponents of other LAMA/LABA combinations (70–95 mL versus LABA) [21, 22]. However, caution should be applied when comparing effect sizes between studies and head-to-head studies are required for more meaningful comparisons.

Both aclidinium/formoterol FDC doses provided clinically meaningful (≥1 unit) improvements in TDI focal score versus placebo with more patients achieving a ≥1-unit improvement. The change in TDI in the placebo group makes comparisons of the change versus placebo difficult; however, the magnitude of change in the placebo group (1.2 units) is the same as that observed in other studies [21, 22] and the changes from baseline of 2.5 and 2.4 units for FDC 400/12 μg and 400/6 μg, respectively, clearly exceed the MCID. The magnitude of improvement was numerically greater than that seen with both monotherapies, although the study was not powered to detect differences between aclidinium/formoterol FDC and monotherapy for TDI or SGRQ. Much of the data for MCID in TDI focal score and SGRQ total score has been reported from placebo-controlled trials, where the treatment effects can be large; for studies investigating the incremental gain from adding one active treatment on top of another, it is likely that the treatment effects will be smaller than the differences between monotherapies and placebo [25].

A ‘Hawthorne effect’ often occurs in COPD trials whereby patients treated with placebo improve by participating in a clinical study [27], particularly in countries where healthcare is not free or easily accessible. However, the SGRQ total score improvement observed in the placebo group (-6.5 units) was considerably larger than that observed in most other COPD clinical trials; a meta-analysis of long-acting bronchodilator COPD trials showed the placebo effect on SGRQ to be typically 2 units, with no studies demonstrating increases of >5 units [28]. The large and mostly unexplained placebo effect that we observed resulted in neither aclidinium/formoterol FDC dose, causing significantly greater SGRQ improvements compared with placebo, despite producing large changes from baseline. However, responder analysis based on the proportion with ≥4-unit improvement did show a benefit with FDC 400/6 μg. Post-hoc analyses showed that ~90% of patients were randomised during winter/spring and treatment continued into the spring/summer period when exacerbation rates are lower [29]; this probably contributed to the low exacerbation rate in the study and large improvement in health status in the placebo arm.

Patients with COPD often report a range of symptoms during normal sleeping hours and on waking [30], and early morning has been reported as the time of day when COPD symptoms are most troublesome [8]. Total daily symptom scores, assessed using the recently validated E-RS questionnaire [12, 13], were significantly lower with aclidinium/formoterol FDC versus placebo and both monotherapies. In addition, night-time and early morning symptoms were improved with aclidinium/formoterol FDC versus placebo and aclidinium. Aclidinium twice-daily has been shown to improve night-time and early morning symptoms [18] and our results suggest that aclidinium/formoterol FDC 400/12 μg provides greater improvements across the whole 24 hours than aclidinium monotherapy.

Both aclidinium/formoterol FDC doses were well tolerated. There was no evidence for additive AEs when combining the two different drugs and no evidence of an increase in AEs at the higher FDC dose. These safety data are consistent with the fact that formoterol is a well-tolerated drug [31] and aclidinium has low systemic exposure, minimising the potential for typical LAMA side effects [32, 33]. Furthermore, the 24-hour Holter-monitoring showed no evidence that aclidinium/formoterol FDC increased ECG abnormalities compared with placebo.

## Conclusions

The twice-daily LAMA/LABA aclidinium/formoterol FDCs had a rapid and sustained effect on lung function, with significant improvements in bronchodilation compared with monotherapy and COPD symptoms compared with placebo. Overall, the benefits seen with aclidinium/formoterol FDC 400/12 μg were greater than those seen with the 400/6 μg dose. Both doses were well tolerated and had similar safety profiles to placebo. These results suggest that aclidinium/formoterol FDC may be an effective new treatment option as a dual bronchodilator for patients with moderate-to-severe COPD.

## Endnote

^a^Registered trademarks of AstraZeneca PLC, Barcelona, Spain; for use as Pressair® within the USA and Genuair® within all other licensed territories.

## Electronic supplementary material

Additional file 1: **Online supplement.** (DOCX 2 MB)

## Abbreviations

AE: Adverse event
BDI: Baseline dyspnoea index
BID: Twice daily
COPD: Chronic obstructive pulmonary disease
ECG: Electrocardiogram
E-RS: EXACT-respiratory symptoms
EXACT: EXAcerbations of Chronic obstructive pulmonary disease Tool
FDC: Fixed-dose combination
FEV_1_: Forced expiratory volume in 1 second
FVC: Forced vital capacity
HCRU: Healthcare resource utilisation
ICS: Inhaled corticosteroids
ITT: Intent-to-treat
LABA: Long-acting β_2_-adrenergic agonist
LAMA: Long-acting muscarinic antagonist
LS: Least squares
MACE: Major adverse cardiovascular event
MCID: Minimal clinically important difference
MMRM: Mixed model for repeated measures
SAE: Serious adverse event
SD: Standard deviation
SGRQ: St George’s respiratory questionnaire
TDI: Transition dyspnoea index
TEAE: Treatment emergent adverse event.
