# Supplementary material for: Efficacy and safety of aclidinium bromide/formoterol fumarate fixed-dose combinations compared with individual components and placebo in patients with COPD (ACLIFORM-COPD): a multicentre, randomised study
Source: BMC Pulm Med. 2014 Nov 18;14:178. doi: 10.1186/1471-2466-14-178 (PMC4273456; doi:10.1186/1471-2466-14-178)
Supplement: Supplementary file 1 — Additional file 1: Online supplement. (DOCX 2 MB) [file 12890_2014_628_MOESM1_ESM.docx]

# Additional File 1 for: Efficacy and safety of aclidinium bromide/formoterol fumarate fixed-dose combinations compared with individual components and placebo in patients with COPD (ACLIFORM-COPD): a multicentre, randomised study

Dave Singh,^1^ Paul W Jones,^2^ Eric D Bateman,^3^ Stephanie Korn,^4^ Cristina Serra,^5^ Eduard Molins,^5^ Cynthia Caracta,^6^ Esther Garcia Gil,^5^ Anne Leselbaum^5^

## Methods

**Study centres**

The study was conducted in 22 countries: Austria, Belgium, Bulgaria, Croatia, Czech Republic, Denmark, Finland, France, Germany, Hungary, Italy, the Netherlands, Poland, Romania, Russia, Slovakia, Spain, Sweden, Ukraine, UK, South Africa and South Korea

**Exclusion criteria and additional concomitant medication**

Exclusion criteria included: history/current diagnosis of asthma; respiratory tract infection or chronic obstructive pulmonary disease (COPD) exacerbation within 6 weeks (3 months if hospitalisation required) pre-screening; clinically relevant respiratory conditions other than COPD; clinically significant cardiovascular conditions; and contraindications to anticholinergics.

Aside from salbutamol and inhaled corticosteroids (ICS), additional permitted medications included oral sustained-release methylxanthines, oxygen therapy (<15 hours/day) and oral or parenteral corticosteroids equivalent to ≤10 mg/day of prednisone or 20 mg every other day, provided treatment was stable ≥4 weeks pre-screening.

**Assessment of E-RS, night-time and early morning symptoms, and exacerbations**

Night-time/early morning symptoms were assessed using a 14-item Night-time and Early Morning Symptoms questionnaire completed every morning. The psychometric qualities of the Night-time and Early Morning Symptoms questionnaires have since been evaluated and final instruments developed [1,2]. Higher E-RS, night-time and early morning symptoms scores indicated more severe symptoms. COPD exacerbations were assessed by the investigator using Healthcare Resource Utilisation (HCRU; an increase of COPD symptoms during ≥2 consecutive days that require a change in COPD treatment) and by the EXACT patient-reported outcomes tool. An EXACT exacerbation was defined as a persistent increase from baseline in total EXACT score of ≥9 points for ≥3 days or ≥12 points for ≥2 days [3,4].

**Statistical analysis of exacerbations**

COPD exacerbations were analysed as both efficacy and safety variables.  Safety analyses (AEs and SAEs) included COPD exacerbations throughout the study, including the follow-up period; efficacy analyses included COPD exacerbations that occurred over the 24 treatment weeks.

The rate of COPD exacerbations per patient per year was analysed by means of a negative binomial regression model including age as a covariate, and treatment group, sex, baseline ICS use, baseline COPD severity and smoking status as factors. However, when the negative binomial model failed to converge, analysis was performed by a Poisson regression model with robust variance estimate using the sandwich method.

## Results

**Change from baseline in FEV_1_ over 3 hours post-dose**

Aclidinium/formoterol fixed-dose combination (FDC) 400/12 µg and 400/6 µg caused significantly greater changes from baseline in forced expiratory volume in 1 second (FEV_1_) at all measured time points over the first 3 hours post-dose at every visit (except Visit 5 [post-dose spirometry not performed]) during the 24-week study compared with placebo (Additional File 1: Figure S1). Improvements in FEV_1_ with aclidinium/formoterol FDCs were observed by 5 minutes post-dose on Day 1 (400/12 µg: 108 mL, 400/6 µg: 100 mL; both p<0.001 vs placebo). Aclidinium and formoterol monotherapies also caused improvements compared with placebo (p<0.05). Additional File 1: Figure S1 shows that improvements in change from baseline in FEV_1_ at all measured time points over the first 3 hours post-dose were significantly greater with aclidinium/formoterol FDC 400/12 µg and FDC 400/6 µg versus either monotherapy during the 24-week study (p<0.05), except at 5 minutes on Day 1 versus formoterol.

**Change from baseline in peak FEV_1_ at all visits**

All active treatments caused significantly greater changes from baseline in peak FEV_1_ at every visit (except Visit 5 [post-dose spirometry not performed]) during the 24-week study compared with placebo (all p<0.001; Week 24 least squares [LS] means: 334 mL, 288 mL, 213 mL, 196 mL and 35 mL for FDC 400/12 µg, FDC 400/6 µg, aclidinium, formoterol and placebo, respectively; Additional File 1: Figure S2). Aclidinium/formoterol FDC 400/12 µg and 400/6 µg caused significantly greater changes from baseline in peak FEV_1_ at all visits compared with both monotherapies (p≤0.001; Additional File 1: Figure S2).

**Change from baseline in normalised FEV_1_ AUC_0–12_ at all visits (12-hour spirometry sub-study)**

FEV_1_ curves for the 12-hour spirometry sub-study [N=366]) are shown in Additional File 1: Figures S3A (Day 1) and Additional File 1: Figure S3B (Week 24). Aclidinium/formoterol FDC 400/12 µg and 400/6 µg provided significantly greater changes from baseline in normalised FEV_1_ AUC_0‑12_ at every time point compared with placebo (LS means: 201 mL and 191 mL, respectively, at Day 1; 220 mL, both doses at Week 12; 221 mL and 189 mL, respectively, at Week 24; all p<0.001). Aclidinium and formoterol monotherapies also caused improvements at every time point compared with placebo (p<0.05). Improvements in change from baseline in FEV_1_ AUC_0–12_ were significantly greater with both aclidinium/formoterol FDC 400/12 µg and 400/6 µg versus formoterol at every time point (LS means 102 mL and 93 mL, respectively, at Day 1 [both p<0.001]; 120 mL, both doses at Week 12 [both p<0.001]; 122 mL and 90 mL, respectively, at Week 24 [both p<0.05]). Compared with aclidinium, significant differences were observed only at Day 1 for aclidinium/formoterol FDC 400/12 µg and 400/6 µg (69 mL and 59 mL, respectively; both p<0.05).

**Change from baseline in E-RS, night-time and early morning symptoms over 24 weeks**

Changes from baseline in overall ER-S scores were -1.66 (-13.4%), -2.48 (-21.6%), ‑2.82 (-25.3), -1.59 (-13.2%) and -1.79 (-15.4%) for placebo, FDC 400/12 µg, FDC 400/6 µg, aclidinium 400 µg and formoterol 12 µg, respectively. The improvements with both FDC doses were significantly greater compared with the monotherapies and placebo (all comparisons p<0.05). Changes from baseline in night-time symptoms scores were -0.17 (-17.2 %), -0.24 (-25.2 %), -0.29 (-30.0 %), -0.15 (-14.7 %) and ‑0.20 (-21.0 %) for placebo, FDC 400/12 µg, FDC 400/6 µg, aclidinium 400 µg and formoterol 12 µg, respectively. Improvements were observed with aclidinium/formoterol versus placebo, with the 400/6 µg dose achieving statistical significance (p<0.01). Both doses of the FDC significantly improved overall night-time symptom severity score versus monotherapy, with the exception of the FDC 400/12 µg dose versus formoterol 12 µg (all p<0.01). Changes from baseline in early morning symptoms scores were -0.12 (-9.6 %), -0.21 (-17.7 %), -0.24 (-20.2 %), ‑0.13 (-10.2 %) and -0.17 (-14.0 %) for placebo, FDC 400/12 µg, FDC 400/6 µg, aclidinium 400 µg and formoterol 12 µg, respectively, with both doses of aclidinium/formoterol FDC achieving statistical significance compared with placebo (both p<0.05) and aclidinium monotherapy (both p<0.01), and FDC 400/6 µg achieving statistical significance versus formoterol (p<0.05).

**TDI and SGRQ responders**

A significantly higher proportion of FDC-treated and monotherapy treated patients had ≥1 unit improvement in TDI focal score at Week 24 versus placebo (Additional File 1: Table S2).

More patients receiving aclidinium/formoterol FDC 400/12 µg or 400/6 µg achieved a clinically meaningful improvement (≥4 unit decrease) in the SGRQ total score compared with placebo at Week 24 (55.3% and 64.2% vs 53.2%, p<0.05 for FDC 400/6 µg vs placebo). Treatment with aclidinium/formoterol 400/6 µg increased the likelihood of patients achieving 4 unit improvement in SGRQ total score versus placebo by 1.77-fold (95% CI: 1.07, 2.95; p<0.05).

#### **Change from baseline in COPD exacerbations**

The rate of exacerbations (any severity) per patient per year was numerically lower with aclidinium/formoterol FDC 400/12 µg (0.26) compared with FDC 400/6 µg (0.29), aclidinium (0.29), formoterol (0.41) and placebo (0.36). Aclidinium/formoterol FDC 400/12 µg and FDC 400/6 µg reduced the rate of exacerbations of any severity by 27% and 20%, respectively, compared with placebo, although these reductions were not significant (Additional File 1: Table S3). The rate of moderate-to-severe exacerbations was also reduced with aclidinium/formoterol FDC 400/12 µg and 400/6 µg by 23% and 15%, respectively; again, these reductions were not statistically significant. Based on EXACT criteria, the rate of events per patient per year was numerically lower with aclidinium/formoterol FDC 400/12 µg (1.09) compared with FDC 400/6 µg (1.28), aclidinium (1.40), formoterol (1.26) and placebo (1.54). A significant reduction in the rate of EXACT events was seen with the higher aclidinium/formoterol FDC dose versus placebo (29%, p<0.05; Additional File 1: Table S3).

**Safety and tolerability**

Most TEAEs were mild or moderate and were not considered to be study treatment related. COPD exacerbation, headache and nasopharyngitis were the most common TEAEs associated with active treatments (≥5% patients overall), although the proportion of patients experiencing COPD exacerbation or headache was lower with the FDCs compared with placebo (Table 3). The most frequently reported SAE was COPD exacerbation, which was higher in the placebo group (2.6%) compared with both FDCs (both 1.0%) or monotherapy (0.3–1.8%) (Table 3).

The incidence of MACE was low and comparable across all study arms (n=3 [0.8%] for aclidinium/formoterol 400/12 µg and formoterol 12 µg; n=2 [0.5%] for aclidinium/formoterol 400/6 µg; and n=1 for aclidinium 400 µg [0.3%] and placebo [0.5%]).

The most common anticholinergic TEAE was oropharyngeal pain, most frequently reported by patients receiving aclidinium/formoterol FDC 400/12 µg (2.6% versus 0.5–1.3% in all other groups including placebo).

In the 24-hour Holter substudy (n=317), non-sustained supraventricular tachycardia was the most frequent observation and was most common in placebo-treated patients (32.1%) versus patients receiving active treatment (20.4–24.6%) (Additional File 1: Table S4).

**Additional File 1: Table S1. Name and address of the central and local IEC in each country**

| **Country** | **Name and address of central IEC** | **Name and address of local IEC** |
| --- | --- | --- |
| Austria | Ethikkommission der Medizinischen, Universität Graz, Auenbruggerplatz 2, 8036 Graz | Ethics committee of the Medical University Graz, Auenbruggerplatz 2, 8036 Graz |
|  |  | Ethics committee of the country Salzburg, Sebastian- Stief-Gasse 2, 5010 Salzburg |
| Belgium | Ethisch Comité UZA, Wilrijkstraat 10, Edegem 2650 | NA |
| Bulgaria | Ethics Committee for Multicenter Trials (ECMT), 5, “Sveta Nedelya” Square, 1000 Sofia | Local Ethics Committee UMHAT Aleksandrovska EAD, 3, Georgi Sofiyski Str. 1431 Sofia |
|  |  | Local Ethics Committee SHATTPD-Ruse EOOD, 1, Aleya Lilia Str. 7002 Ruse |
|  |  | Local Ethics Committee SHATPFD-Sofia District EOOD, 309, “Slivnitsa” Blvd, 1234 Sofia |
|  |  | Local Ethics Committee DCC Sveta Anna EOOD, 1, Dimitar Mollov Str. 1709 Sofia |
|  |  | Local Ethics Committee DCC “Akta Medika” EOOD, 60, “Nikola Petkov” Str., 5400 Sevlievo |
| Croatia | Agency for Medicinal Product and Medical Devices of Croatia,  Central Ethics Committee, Ksaverska cesta 4, Zagreb, 10000 | NA |
| Czech Republic | Multicentricka eticka komise Fakultni nemocnice u sv. Anny v Brne, Vystavni 17/19, Brno, 656 | NA |
| Denmark | De Videnskabsetiske Komitéer for Region Hovedstaden, Kongens Vænge 2, 3400 Hillerød | NA |
| Finland | Keski-Suomen sairaanhoitopiiri, Eettinen toimikunta, Sairaan hoitopiirin toimisto Rak. 6/2, Keskussairaalantie 19, 40620 Jyväskylä | NA |
| France | Comité de Protection des Personnes Sud Ouest et Outre Mer III, Place Amélie Raba-Léon, Groupe Hospitalier Pellegrin – Service de Pharmacologie Clinique, Bât 1 A, Bordeaux cedex, 33076 | NA |
| Germany | Landesärztekammer Rheinland-Pfalz, Postfach 29 26,  55019 Mainz55019 Mainz | Landesamt für Gesundheit und Soziales Berlin, Geschäftsstelle der Ethik-Kommission des Landes Berlin, Fehrbelliner Platz 1, 10707 Berlin |
|  |  | Ethik-Kommission des Landes Sachsen-Anhalt, Geschäftsstelle, Kühnauer Str. 70, 06846 Dessau-Roßlau |
|  |  | Ethik-Kommission der Ärztekammer Westfalen-Lippe und der Medizinischen Fakultät der Westfälischen Wilhelms-Universität Münster, Gartenstr. 210–214, 48147 Münster |
|  |  | Landesärztekammer Hessen, Ethikkommission, Im Vogelsgesang 3, 60488 Frankfurt am Main |
|  |  | Sächsische Landesärztekammer, Ethikkommission, Schützenhöhe 16–18, 01099 Dresden |
|  |  | Ärztekammer Hamburg, Ethikkommission, Humboldtstr. 67a, 22083 Hamburg |
|  |  | Landesärztekammer Rheinland-Pfalz, Ethikkommission, Deutschhausplatz 3, 55116 Mainz |
|  |  | Ärztekammer Nordrhein, Ethikkommission, Tersteegenstr. 9, 40474 Düsseldorf |
|  |  | Ethikkommission der Medizinischen Fakultät der Universität Rostock, Institut für Rechtsmedizin, St. Georg-Str. 108 |
|  |  | Ärztekammer Niedersachsen, Ethikkommission, Berliner Allee 20, 30175 Hannover |
|  |  | Bayerische Landesärztekammer, Ethikkommission, Mühlbaurstr. 16, 81677 München |
| Hungary | Egészségügyi Tudományos Tanács Klinikai Farmakológiai Etikai Bizottsága, Arany J. u. 6–8, Budapest, H-1051 | NA |
| Italy | Comitato Etico Locale per la Sperimentazione Clinica dei Medicinali dell'Azienda Ospidaliero ra Universitaria ria Senese di Siena, c/o Farmacia AOUS Viale Bracci, Siena, 53100 | Comitato Etico Unico per la Provincia di Parma, Via Gramsci, 14, Parma, 43100 |
|  |  | Comitato per la Sperimentazione Clinica Medicinali dell'Azienda Ospedaliero Universitaria Pisana di Pisa, Via Roma 67, Pisa, 56100 |
|  |  | Comitato Etico Locale per la Sperimentazione Clinica dei Medicinali dell'Azienda Ospidaliero ra Universitaria ria Senese di Siena, c/o Farmacia AOUS Viale Bracci, Siena, 53100 |
|  |  | Comitato Etico dell'Azienda Ospedaliero Universiitaria S.Martino di Genova, Largo Rosanna Benzi, 10, Genova, 16132 |
| Republic of Korea | NA | IRB of Ewha Womans University Mokdong Hospital, 1071, Anyangcheon-ro, YangCheon‑Ku, Seoul, 158-710 |
|  |  | IRB of Korea University Anam Hospital, 73 Inchon-ro, Seongbuk-Gu, Seoul, 136-705 |
|  |  | IRB of Hallym University Sacred Heart Hospital, 896 Pyeongchon-dong, Dongan‑gu, Anyang-si, 431-070 |
|  |  | IRB of The Catholic University of Korea, Seoul St.Mary's Hospital 222 Banpo-Daero, Seocho‑gu, Seoul, 137-701 |
|  |  | IRB of Soonchunhyang University Bucheon Hospital, 1174 Joong-dong, Wonmi-gu, Buchon-si, 420-767 |
|  |  | IRB of Korea University Guro Hospital, 148 Gurodong-ro, Guro-Gu, Seoul, 152-703 |
|  |  | IRB of Yonsei University Wonju Christian Hospital, 20 Ilsan-Ro, Wonju-Si, 220-701 |
|  |  | IRB of Seoul National Hospital, 101 Daehak-ro Jongno-gu, Seoul, 110-744 |
| Netherlands | METC Catharina ziekenhuis, Michelangelo laan 2, Eindhoven, 5623 EJ | NA |
| Poland | Komisja Bioetyczna przy Instytucie Gruzlicy I Chorob Pluc, ul. Plocka 26, Warszawa, 01-138 | NA |
| Romania | Comisia Nationala de Etica, Str. Aviator Sanatescu Nr. 48, Bucuresti, Sector 1,01 | NA |
| Russian Federation | Ethical Council at the MoH of RF, 3 Rakhmanovsky Pereulok, Moscow, 127994 | LEC at City Clinical Hospital #23 n.a. Medsantrud, 11, Yauzskaya str, Moscow, 109240 |
|  |  | LEC FA of HealthCare andSD StP Reumatology Clinical Hosp.#25, 47, Piskarevsky prospect, St. Petersburg, 195067 |
|  |  | LEC at SRI of Therapy of Siberian branch of Russian Academy of Medical Sciences, 175/1, B.Bogatkova str, Novosibirsk, 630089 |
|  |  | LEC at StP SMU n.a. acad. I.P. Pavlov, 10, Rentgena str.Saint-Petersburg, 197101 |
|  |  | LEC at FSd ESMC of presed. RF City Hospital #17, Volynskaya, 7, Moscow, 119620 |
| Slovakia | Fakultná nemocnica s poliklinikou F.D. Roosevelta, Nám. L. Svobodu 1, 975 17 Banská Bystrica | Nitriansky samosprávny kraj Úrad Nitrianskeho samosprávneho kraja Etická komisia, Štefánikova tr. 69, 949 01 Nitra |
|  |  | Bratislavský samosprávny kraj, Úrad samosprávneho kraja, Etická komisia, Sabinovská 16, P.O. Box 106, 820 05 Bratislava 25 |
|  |  | Košický samosprávny kraj, Úrad Košického samosprávneho kraja Etická komisia, Námestie Maratónu mieru 1, 042 66 Košice |
|  |  | NsP Sv. Jakuba, n.o., Bardejov, ul. Sv. Jakuba 21, 085 01 Bardejov |
|  |  | Fakultná nemocnica s poliklinikou F.D. Roosevelta, Nám. L. Svobodu 1, 975 17 Banská Bystrica |
|  |  | EK-Narodny ustav TBC, plucnych chorob a hrudnikovej chirurgie, Vysne Hagy, Vysne Hagy, 05984 |
|  |  | Nitriansky samosprávny kraj Úrad Nitrianskeho samosprávneho kraja Etická komisia, Štefánikova tr. 69, 949 01 Nitra |
| South Africa | NA | Pharma Ethics, 123 Amcor Road, Lyttelton Manor 0157 |
|  |  | University of Cape Town Ethics Committee, Faculty of Health Sciences Research EC, E52‑24 Old Main Building, Groote Schuur Hospital, Observatory, Cape Town, 7925 |
|  |  | University of Stellenbosch Ethics Committee, Faculty of Health Sciences, Francie van Zijl Drive, Cape Town, 7505 |
|  |  | Pharma Ethics, 123 Amcor Road, Lyttelton Manor 0157 |
|  |  | University of Pretoria, Research Ethics Committe, 31 Bophelo Road, HW Snyman South Building, Level 2, Room 2:34, Pretoria, Gauteng, 0001 |
| Spain | CEIC Hospital Universitario Puerta de Hierro Majadahonda, Planta 1ª, Pasillo unidades, administrativas de servicios,  c/ Manuel de Falla, 1, Majadahonda, 28222 | CEIC Institut Municipal d’Assistència Sanitària, C/ Doctor Aiguader, 88 Edifici PRBB Barcelona, 08003 |
|  |  | Comité Ético de Investigación Clínica de Asturias, C/ Celestino Villamil, s/n Oviedo, 33006 |
|  |  | CEIC Hospital virgen de la Macarena, Dirección: Avda. Dr. Fedriani, 3 – Unidad de Investigación 2ª planta Sevilla, 41071 |
|  |  | CEIC del Complejo Hospitalario de Cáceres, Avda. Pablo Naranjo s/n Caceres, 10003 |
|  |  | CEIC del IDIAP Jordi Gol i Gurina, Gran Via de les Corts Catalanes, 587 atico Barcelona, 08007 |
|  |  | CEIC Hospital Germans Trias i Pujol, Ctra. Canyet, s/n Badalona, 08916, |
|  |  | CEIC Hospital general Carlos Haya, Avda. Carlos Haya, s/n Málaga, 29010 |
| Sweden | Regionala etikprövningsnämnden i Lund, Box 133, Östra Vallgatan, 14/Östervångsvägen 1, Lund, 22100 | NA |
| Ukraine | Central Ethics Commission of the Ministry of Health of Ukraine, 5, Narodnogo Opolchennya St., Kyiv, 03680 | LEC "Kharkiv City Clinical Hospital # 13", 137, Gagarin Av. Kharkiv, 61035 |
|  |  | LEC SI "National Institute of Phthisiology and Pulmonology named F.G.Yanovskyy of AMS of Ukr, 10, Amosova Str., Kyiv, 03680 |
| United Kingdom | NRES Committee North West, 3rd Floor, Barlow House, 4 Minshull Street, Manchester, M1 3DZ | NRES Committee North West, 3rd Floor Barlow House, 4 Minshull Street, Manchester, M1 3DZ |
|  |  | Bart ‘s Health NHS Trust, Joint Research and Development Office, Queen Mary Innovation Centre, 5 Walden Street, London, E1 2EF |
|  |  | The Royal Wolverhampton Hospitals NHS Trust, Research & Development Directorate, The Chestnuts, Wolverhampton, West Midlands, WV10 0QP |
|  |  | Cambridge University Hospitals NHS Foundation Trust, Addenbrookes Hospital, Hills Road, Cambridge, CB2 0QQ |
|  |  | Hull & East Yorkshire Hospitals NHS Trust, Research & Development Department, 2nd Floor Daisy Building, Castle Hill Hospital, Castle Road, Cottingham, East Yorkshire, HU16 5JQ |
|  |  | Bradford Teaching Hospitals NHS Foundation Trust, Bradford Royal Infirmary, Room 6, Pharmacy Stores, Gate 7, Smith Lane, Bradford West Yorkshire, BD9 6RJ |
|  |  | NRES Committee West Midlands, Prospect House, Fishing Line Road, Enfield, Redditch, B97 6EW |
|  |  | NRES Committee South Central, Building L27, University of Reading, London Road, Reading, RG1 5AQ |
|  |  | North Tees & Hartlepool NHS Foundation Trust – R&D, Hardwick Road, Stockton on Tees, TS19 8PE |
|  |  | Wirral University Teaching Hospital NHS Foundation Trust, Arrowe Park Hospital, Arrowe Park Road, Upton, Wirral, CH49 5PE |
|  |  | NHS Royal Victoria Infirmary, Queen Victoria Road, Newcastle Upon Tyne, Newcastle, NE1 4LP |

IEC, Independent Ethics Committee

**Additional File 1: Table S2. Mean treatment differences between all active treatments and placebo, and between FDC and its monotherapy components in TDI focal score, TDI responders and SGRQ total score at Week 24 (ITT population).**

| **Parameter** | **Placebo** | **FDC 400/12 µg** | **FDC 400/6 µg** | **Aclidinium 400 µg** | **Formoterol 12 µg** |
| --- | --- | --- | --- | --- | --- |
| TDI focal score, LS mean | 1.22 (0.74, 1.69) | 2.51 (2.19, 2.83) | 2.38 (2.05, 2.70) | 2.11 (1.79, 2.44) | 2.06 (1.74, 2.39) |
| Difference vs placebo | - | 1.29 (0.73, 1.86)^***^ | 1.16 (0.59, 1.73)^***^ | 0.90 (0.33, 1.47)^**^ | 0.85 (0.28, 1.42)^**^ |
| Difference vs aclidinium 400 µg | - | 0.40 (-0.05, 0.85) | 0.27 (-0.19, 0.72) | - | - |
| Difference vs formoterol 12 µg | - | 0.45 (-0.00, 0.90) | 0.31 (-0.14, 0.77) | - | - |
| TDI responders (%) | 45.5 | 64.8 | 63.7 | 56.5 | 61.3 |
| OR vs placebo | - | 2.54 (1.57, 4.10)^***^ | 2.57 (1.59, 4.16)^***^ | 1.79 (1.11, 2.89)^*^ | 2.14 (1.32, 3.45)^**^ |
| OR vs aclidinium 400 µg | - | 1.42 (0.97, 2.07) | 1.43 (0.98, 2.10) | - | - |
| OR vs formoterol 12 µg | - | 1.19 (0.81, 1.74) | 1.20 (0.82, 1.77) | - | - |
| SGRQ total score, LS mean | -6.51 (-8.53, -4.49) | -7.16 (-8.54, -5.79) | -8.34 (-9.72, -6.96) | -5.80 (-7.19, -4.41) | -5.58 (-6.96, -4.20) |
| Difference vs placebo | - | -0.65 (-3.08, 1.78) | -1.83 (-4.26, 0.60) | 0.71 (-1.73, 3.15) | 0.93 (-1.50, 3.37) |
| Difference vs aclidinium 400 µg | - | -1.36 (-3.30, 0.58) | -2.54 (-4.48, -0.59)^‡^ | - | - |
| Difference vs formoterol 12 µg | - | -1.59 (-3.52, 0.35) | -2.76 (-4.70, -0.82)^††^ | - | - |

Data are presented as LS means or OR (95% CI). ^*^p<0.05; ^**^p<0.01; ^***^p<0.001 vs placebo; ^‡^p<0.05 vs aclidinium. ^††^p<0.01 vs formoterol.

FDC, aclidinium/formoterol fixed-dose combination; ITT, intention-to-treat; LS, least squares; OR, odds ratio; TDI, Transition Dyspnoea Index; SGRQ, St George’s Respiratory Questionnaire.

**Additional File 1: Table S3. Rate of COPD exacerbations (any severity) per patient per year based on HCRU and EXACT definitions (ITT exacerbation population).**

| **Parameter** | **Placebo** | **FDC  400/12 µg** | **FDC  400/6 µg** | **Aclidinium 400 µg** | **Formoterol 12 µg** |
| --- | --- | --- | --- | --- | --- |
| HCRU rate | 0.36 (0.23, 0.54) | 0.26 (0.19, 0.36) | 0.29 (0.21, 0.39) | 0.29 (0.21, 0.40) | 0.41 (0.31, 0.54) |
| RR vs placebo | - | 0.73 (0.4, 1.2) | 0.80 (0.5, 1.4) | 0.82 (0.5, 1.4) | 1.15 (0.7, 1.9) |
| RR vs aclidinium 400 µg | - | 0.89 (0.6, 1.4) | 0.98 (0.6, 1.5) | - | - |
| RR vs formoterol 12 µg | - | 0.64 (0.4, 1.0)^†^ | 0.70 (0.5, 1.1) | - | - |
| EXACT rate | 1.54  (1.2, 1.9) | 1.09 (0.9, 1.3) | 1.28  (1.1, 1.5) | 1.40 (1.2, 1.6) | 1.26  (1.1, 1.5) |
| RR vs placebo | - | 0.71 (0.5, 0.9)^*^ | 0.83 (0.6, 1.1) | 0.91 (0.7, 1.2) | 0.82 (0.6, 1.1) |
| RR vs aclidinium 400 µg | - | 0.78 (0.6, 1.0)^‡^ | 0.91 (0.7, 1.1) | - | - |
| RR vs formoterol 12 µg | - | 0.86 (0.7, 1.1) | 1.01 (0.8, 1.3) | - | - |

Data are presented as rate or RR (95% CI). ^*^p<0.05 vs placebo; COPD, chronic obstructive pulmonary disease; FDC, aclidinium/formoterol fixed-dose combination; EXACT, Exacerbations of Chronic Pulmonary Disease Tool (defined as a persistent increase from baseline in total EXACT score of ≥9 points for ≥3 days or ≥12 points for ≥2 days); HCRU, Healthcare Resource Utilisation (defined as an increase of COPD symptoms during ≥2 consecutive days that require a change in COPD treatment); ITT, intention-to-treat; RR, rate ratio.

| **Additional File 1: Table S4. Observations from 24-hour 12-lead Holter recordings present at Week 24 but not at baseline (Safety population sub-study).** | | | | | |
| --- | --- | --- | --- | --- | --- |
| **Observation, n/N (%)** | **Placebo (N=37)** | **FDC 400/12 µg (N=69)** | **FDC 400/6 µg (N=69)** | **Aclidinium 400 µg (N=71)** | **Formoterol 12 µg (N=71)** |
| Non-sustained supraventricular tachycardia | 3/28 (10.7) | 4/59 (6.8) | 4/58 (6.9) | 2/54 (3.7) | 7/61 (11.5) |
| Frequent VPCs | 4/28 (14.3) | 4/59 (6.8) | 7/58 (12.1) | 6/54 (11.1) | 9/61 (14.8) |
| Torsades de Pointes | 0/28 (0.0) | 0/59 (0.0) | 0/58 (0.0) | 0/54 (0.0) | 1/61 (1.6) |
| Non-sustained ventricular tachycardia | 9/28 (32.1) | 13/59 (22.0) | 13/58 (22.4) | 11/54 (20.4) | 15/61 (24.6) |
| Atrial fibrillation | 0/28 (0.0) | 0/59 (0.0) | 1/58 (1.7) | 1/54 (1.9) | 1/61 (1.6) |
| Atrial flutter | 0/28 (0·0) | 0/59 (0.0) | 1/58 (1.7) | 0/54 (0.0) | 0/61 (0.0) |
| Mobitz I (Wenckebach) 2^nd^ degree AV block | 0/28 (0.0) | 1/59 (1.7) | 0/58 (0.0) | 0/54 (0.0) | 0/61 (0.0) |
| RR interval >2.0 seconds | 1/28 (3.6) | 0/59 (0.0) | 0/58 (0.0) | 0/54 (0.0) | 1/61 (1.6) |
| Bradycardia | 0/28 (0.0) | 1/59 (1.7) | 0/58 (0.0) | 3/54 (5.6) | 0/61 (0.0) |
| Intermittent ectopic atrial rhythm | 0/28 (0.0) | 1/59 (1.7) | 0/58 (0.0) | 0/54 (0.0) | 0/61 (0.0) |
| Intermittent junctional rhythm | 0/28 (0.0) | 2/59 (3.4) | 0/58 (0.0) | 0/54 (0.0) | 0/61 (0.0) |

n/N refers to the number of patients with the finding (n) divided by the number evaluable at the timepoint (N).
AV, atrioventricular; FDC, aclidinium/formoterol fixed-dose combination; RR, Duration in milliseconds between two R peaks of two consecutive QRS complexes; VPC, ventricular premature complexes.

**Additional File 1: Figure S1. Change from baseline in FEV_1_ over 3 hours post-morning dose on (A) Day 1 and at (B) Week 24 (ITT population).**


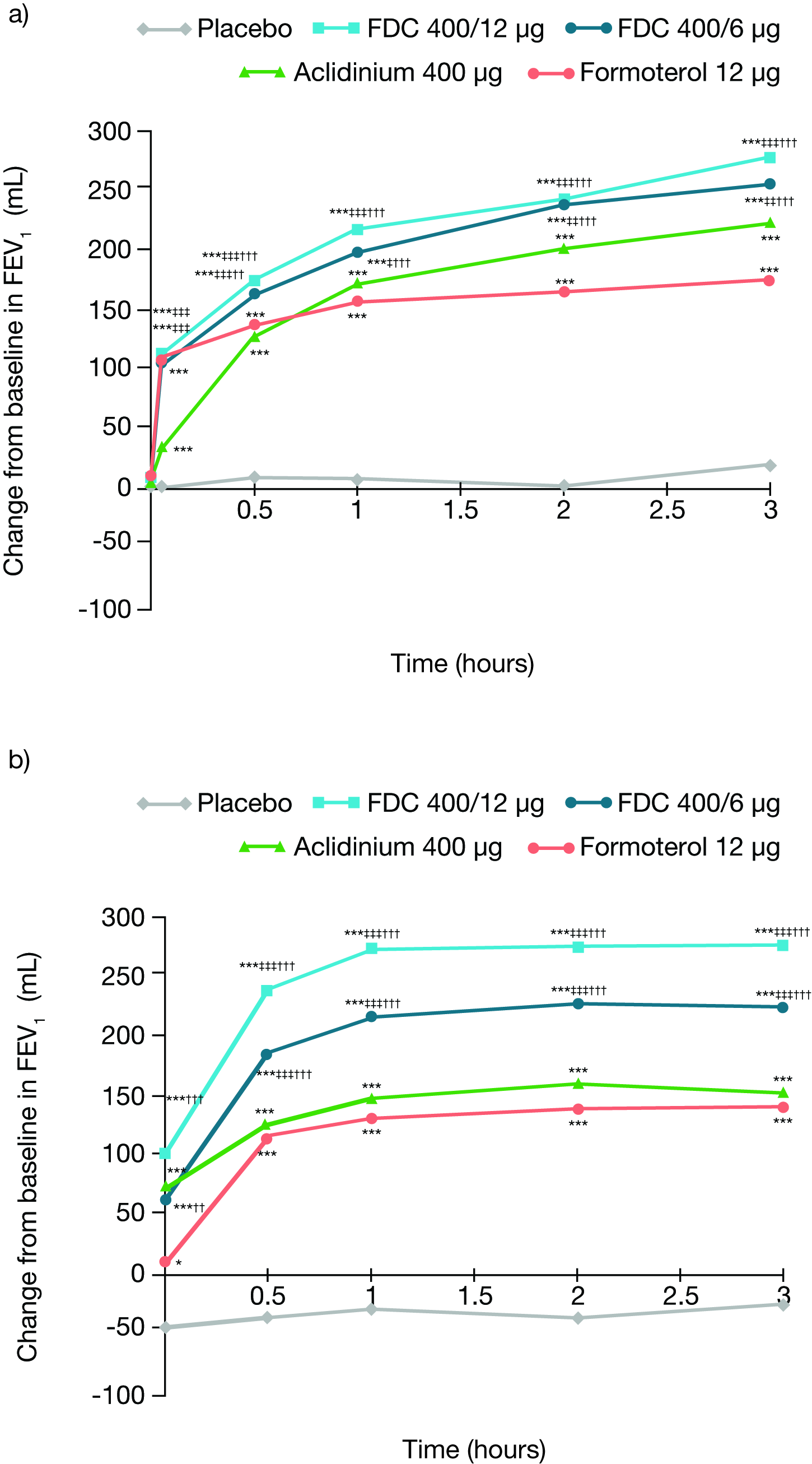


Data are presented as least squares means.
^*^p<0.05, ^***^p<0.001 vs placebo; ^‡^p<0.05, ^‡‡^p<0.01, ^‡‡‡^p≤0.001 vs aclidinium; ^††^p<0.01, ^†††^p<0.001 vs formoterol.
FDC, fixed dose combination of aclidinium/formoterol; FEV_1_, forced expiratory volume in 1 second; ITT, intent-to-treat

**Additional File 1: Figure S2. Mean change from baseline in peak FEV_1_ at all treatment visits (ITT population).**


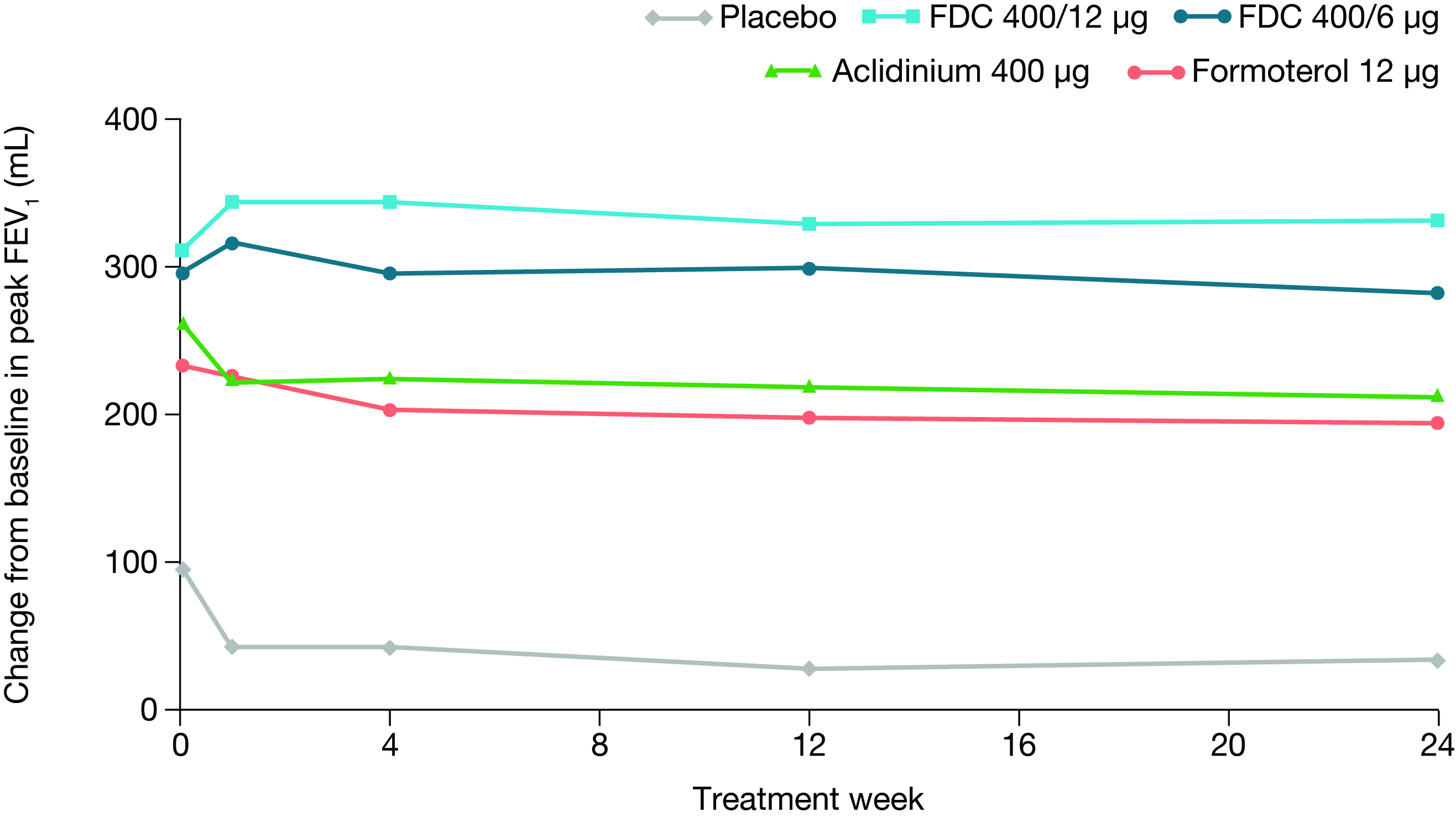


Data are presented as least squares means.
p≤0.001 for all active treatments vs placebo and FDC 400/6 and 400/12 µg vs aclidinium 400 µg and formoterol 12 µg.
FDC, aclidinium/formoterol fixed-dose combination; FEV_1_, forced expiratory volume in 1 second; ITT, intent-to-treat

**Additional File 1: Figure S3. Change from baseline in FEV_1_ over 12 hours post-morning dose on (A) Day 1 and at (B) Week 24 (12-hour spirometry sub-study).**


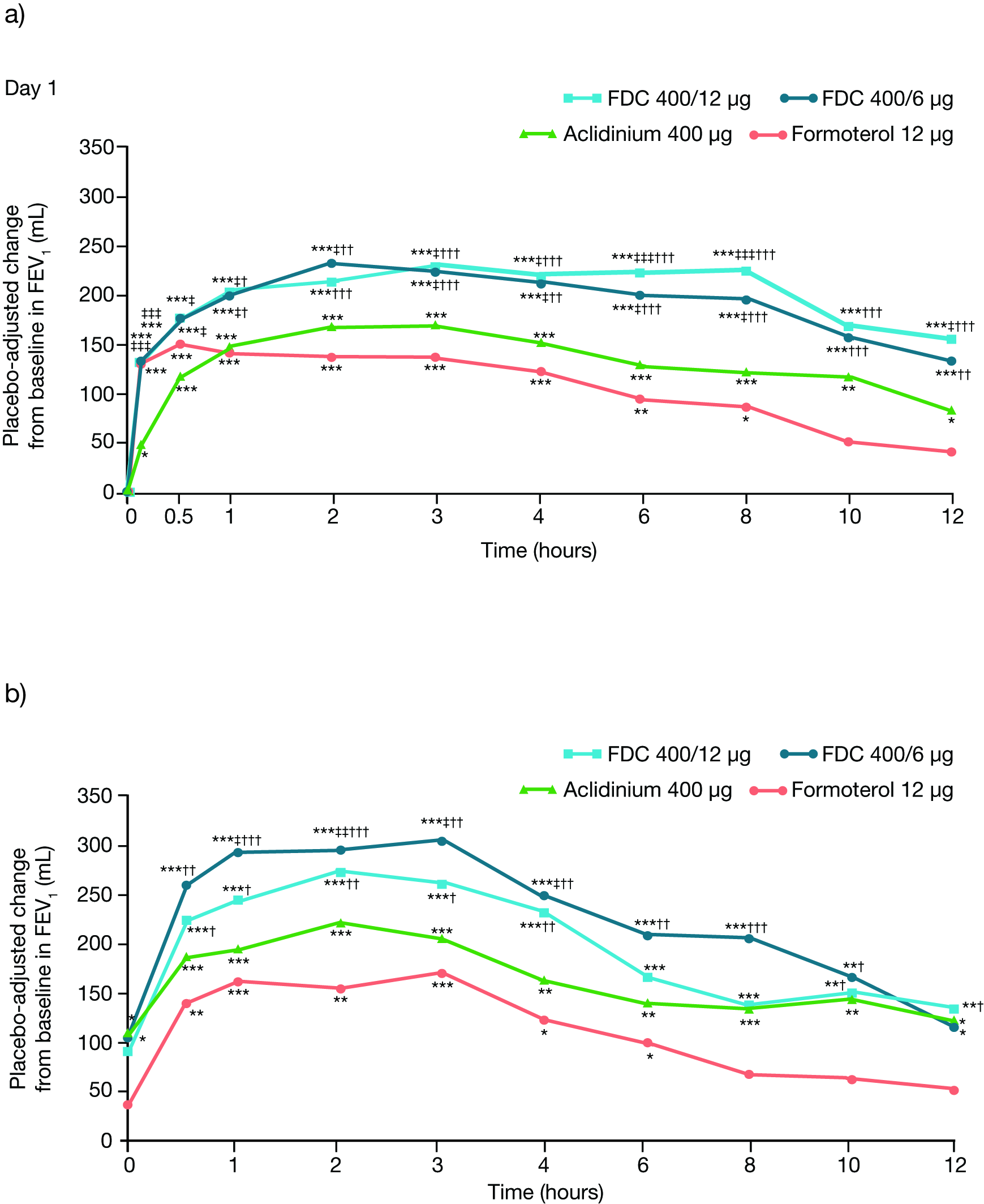


Data are presented as least squares mean differences from placebo.
^*^p<0.05; ^**^p<0.01; ^***^p≤0.001 vs placebo; ^‡^p<0.05, ^‡‡^p<0.01; ^‡‡‡^p<0.001 vs aclidinium; ^†^p<0.05, ^††^p<0.01, ^†††^p≤0.001 vs formoterol
FDC, aclidinium/formoterol fixed-dose combination; FEV_1_, forced expiratory volume in 1 second

**References**

1. Mocarski M, Hareendran A, Jen MH, Zaiser E, Make B: **Evaluation of the psychometric properties of the early morning symptoms of COPD instrument (EMSCI)** **[abstract]**. Presented at the International Society for Pharmacoeconomic and Outcomes Research, May 31-June 4, 2014.

2. Mocarski M, Hareendran A, Jen MH, Zaiser E, Make B: **Evaluation of the psychometric properties of the nighttime symptoms of COPD instrument (NiSCI)** **[abstract]**. Presented at the American Thoracic Society International Conference, San Diego, California, USA, May 16-21, 2014.

3. EXACT-PRO Initiative: **The Exacerbations of Chronic pulmonary disease Tool (EXACT) User Manual Version 4.0** [http://www.exactproinitiative.com/].

4. Leidy NK, Wilcox TK, Jones PW, Roberts L, Powers JH, Sethi S: **Standardizing measurement of chronic obstructive pulmonary disease exacerbations: reliability and validity of a patient-reported diary**. *Am J Respir Crit Care Med* 2011, **183:**323–329.
